# Circadian clock dysfunction in Parkinson’s disease: mechanisms, consequences, and therapeutic strategy

**DOI:** 10.1038/s41531-025-01009-9

**Published:** 2025-07-14

**Authors:** Müge Yalçin, Valentina Grande, Tiago Fleming Outeiro, Angela Relógio

**Affiliations:** 1 Institute for Systems Medicine and Faculty of Human Medicine, MSH Medical School Hamburg, Hamburg, Germany; 2 Department of Experimental Neurodegeneration, Center for Biostructural Imaging of Neurodegeneration, University Medical Center Göttingen, Göttingen, Germany; 3 Max Planck Institute for Multidisciplinary Sciences, Göttingen, Germany; 4 Translational and Clinical Research Institute, Faculty of Medical Sciences, Newcastle University, Newcastle Upon Tyne, UK; 5 Deutsches Zentrum für Neurodegenerative Erkrankungen (DZNE), Göttingen, Germany

**Keywords:** Molecular biology, Translational research

## Abstract

Parkinson’s Disease (PD) is a prevalent neurodegenerative disorder characterized by the progressive loss of dopaminergic neurons in the substantia nigra. This leads to hallmark motor features that include bradykinesia, resting tremor, rigidity, and postural instability, alongside with a range of non-motor symptoms including sleep disturbances, mood disorders, and cognitive decline. As global life expectancy rises, the prevalence of PD is expected to continue to increase, highlighting the urgent need for effective therapeutic strategies. Despite tremendous advances in our understanding of disease-associated mechanisms, we still do not fully understand the aetiology of PD. Emerging evidence points to the circadian clock—a system that regulates physiological processes such as sleep-wake cycles and hormone release—as a critical factor in PD pathophysiology. Disruptions in circadian rhythms (CR) are common in PD patients and may exacerbate both motor and non-motor symptoms, potentially influencing the progression of neurodegeneration. This review examines the global impact of PD within the context of an aging population, delves into the molecular underpinnings of the disease, and explores the role of CR in PD. We summarize and discuss key reports on molecular links between PD and CR using unbiased systematic search strategies to cover a broad literature. Finally, we discuss the potential of chronotherapy, the alignment of treatment with the body’s natural rhythms, as a personalized approach in PD management, aiming to improve treatment efficacy and patient quality of life. Understanding the interplay between circadian biology and PD could pave the way for innovative, personalized therapeutic strategies.

## Introduction

Parkinson’s Disease (PD) is a chronic and progressive neurodegenerative disorder characterized by the degeneration of dopaminergic neurons in the substantia nigra, leading to motor symptoms that include bradykinesia, resting tremor, rigidity, and postural instability (Box 1). Nonmotor symptoms, including sleep disturbances, mood disorders, and cognitive decline, further complicate the disease’s clinical presentation^1^. PD is estimated to affect over nine million people worldwide, and is the second most common neurodegenerative disease after Alzheimer’s disease (AD)^2^. PD is an age-associated disorder and, as such, its prevalence is increasing significantly due to the overall ageing trends of the human population. Moreover, PD is a progressive disorder leading to increasing disability that requires exceptional efforts from caregivers and, therefore, has a tremendous socio-economic impact^3^. In 2017, in U.S. alone about one million individuals were diagnosed with PD for an estimated economic burden of $51.9 billion^4^.

In addition to the loss of dopaminergic neurons, another pathological hallmark of PD is the accumulation of Lewy bodies and Lewy neurites, neuronal cytoplasmic inclusions enriched in alpha-synuclein (aSyn), a protein of 140 amino acids that is abundant in the brain, and becomes insoluble for reasons we do not fully understand^5^.

Although most forms of PD are sporadic, the identification of genetic factors associated with PD has enabled significant progress in our understanding of the molecular mechanisms involved^6,7^. Monogenic forms of PD, caused by a mutation in a single gene, account for only 3–5% of cases. However, a recent large-observational study involving over 12,000 patients, suggested that a genetic contribution—including both monogenic mutations and risk variants such as those in *GBA1*—can be identified in up to 15% of individuals with PD^8^. These findings underscore the relevance of genetic testing in clinical settings, which can support informed decisions for a variety of applications from diagnosis to prognosis of PD, and accordingly the development of targeted therapeutic approaches. The effect of mutations in PD-associated genes range from being “fully penetrant” (like *SNCA* triplications or missense variants causing monogenic PD forms), to conferring a “strong predisposition” to the disease (as *SNCA* duplications, or variants in *LRRK2*, *VPS35*, and *CHCHD2*), or to variants causing “medium predisposition”^6^. To date, over 200 PD-associated genes have been discovered which can interact with other risk factors such as ageing and environmental factors in the development of the pathology^7^. Among the environmental factors, exposure to pesticides such as paraquat or rotenone, or to metals such as iron and manganese, seem to considerably increase the incidence of PD, especially in those more directly exposed to these substances^9–12^.

In understanding the pathogenesis of PD, extensive research has focused on the molecular mechanisms underlying neuronal death and dysfunction. Oxidative stress, mitochondrial dysfunction, and protein misfolding are central to the disease’s molecular pathology^13^. The accumulation of aSyn is hypothesized to contribute to neuronal dysfunction, toxicity, and cell death. These molecular insights have been instrumental in identifying potential therapeutic targets, though effective disease-modifying treatments remain elusive.

Emerging evidence suggests that the circadian clock, the body’s intrinsic timekeeping system, may play a critical role in the pathophysiology of PD. Circadian rhythms (CR), which regulate a wide array of physiological processes, including sleep-wake cycles, hormone release, and metabolic functions, are disrupted in PD patients^14,15^. This disruption not only exacerbates the motor and nonmotor symptoms of PD but may also influence the progression of neurodegeneration. Understanding the link between circadian rhythms and PD could reveal therapeutic strategies that align treatment with the body’s natural rhythms, potentially improving outcomes and quality of life for patients.

The management of PD has traditionally focused on symptomatic relief through pharmacological and surgical interventions. Levodopa (Box 1) remains the gold-standard for the management of motor symptoms, while deep brain stimulation (DBS) is a surgical intervention that offers benefits for patients with advanced disease^16^. However, these treatments do not halt disease progression, and their effectiveness diminishes over time due to complications such as dyskinesias and motor fluctuations. As such, there is a pressing need for innovative approaches that not only alleviate symptoms but also modify the disease course, as well as identifying disease markers for early detection and monitoring.

One promising area of research is the exploration of CR in the management of PD. Given the pervasive influence of circadian clocks on biological functions, optimizing the timing of pharmacological interventions, physical therapy, and lifestyle modifications in accordance with circadian rhythms could enhance treatment efficacy and mitigate side effects. Chronotherapy, the alignment of treatment with the body’s natural rhythms, has shown potential in other chronic diseases such as hypertension^17^, asthma^18^ and could be a valuable strategy in PD management. Moreover, improving sleep and circadian function in PD patients may alleviate some of the nonmotor symptoms, such as depression and cognitive impairment^19^, thereby improving overall patient well-being.

In this review, we cover a wide range of potential medical-related applications of CR—spanning from its use as a biomarker, diagnostic or therapeutic approach while combining insights across cellular or animal models, and humans, with a particular focus on the PD field. By integrating such a broad range of aspects, we aimed to provide a deeper understanding of the connection between PD and CR abnormalities, offering insights that can guide future research and clinical applications, making this review a valuable resource for researchers and clinicians. We discuss the role of the circadian clock in PD, and whether disruptions in CR are merely a consequence of the disease or play a contributory role in its pathogenesis. Additionally, we provide a comprehensive literature summary for each key molecular mechanism associated with PD and connect them to the circadian clock. To avoid biases on our search, we used defined Medical Subject Headings (MeSH) terms and synonym keywords in the field of CR and PD, which allowed us to cover the entire literature and to summary and discuss here the relevant reports for our review. Finally, we will discuss the current state of PD management and the potential benefits of incorporating CR considerations into pharmacological and non-pharmacological therapeutic strategies. Understanding these intersections between time, molecular mechanisms of neurodegeneration, and therapy could pave the way for more personalized and effective treatment approaches for PD patients.

Box 1 Glossary of key terms related to Parkinson’s disease

**Table:** 

**Term**	**Definition**
Bradykinesia	Reduction/slowdown in voluntary movements
Carbidopa	Dopa decarboxylase inhibitor drug used in combination with levodopa for the symptomatic treatment of Parkinson's disease
Cingulate cortex	A subset of “limbic cortex” located in the medial walls of the cerebral hemispheres
COMT inhibitors	Drugs inhibiting the enzyme catechol-*O*-methyltransferase
Dopamine	Endogenous neurotransmitter of the catecholamine family. In PD, low levels of dopamine cause motor impairment
Dopamine agonists	Drugs that activate dopamine receptors
Dopaminergic neurons	Neurons of the midbrain constituting the main source of dopamine (DA) in the mammalian central nervous system
Dopaminergic treatments	Treatments with drugs activating dopamine receptors
Frontal cortex	The neocortex anterior to the motor somatosensory–cortex border
Gait difficulty	Parkinsonian gait is usually characterized by small, shuffling steps and by the difficulty of picking up the feet
Hippocampus	A bilaminar gray matter structure located medially in the temporal lobe, affected by PD in stage 4 and responsible for cognitive deficits
Sporadic PD	PD with unknown cause
Levodopa (L-3,4-Dioxyphenylalanine, L-DOPA)	Intermediate amino acid in the dopamine biosynthetic pathway used to treat Parkinson's disease
Monoamine oxidase type B (MAO-B) inhibitors	B-type monoamine oxidase blockers, they make more dopamine available to treat PD symptoms
6-OHDA or 6-hydroxydopamine	A drug used to induce neurodegeneration of the nigrostriatal system
*Parkin RBR E3 ubiquitin protein ligase* (*PRKN*)	Ubiquitin E3 ligase that, mutated, constitutes the second most common cause of PD
Postural instability	The incapacity to maintain balance in dynamic and static conditions
Resting tremor	Tremor characteristic of PD, occurring mainly at rest
Substantia nigra pars compacta (SNc)	It is a major sub-region of the substantia nigra, made up of dopaminergic neurons which, degenerated or malfunctioned, are associated with PD

## The circadian clock and its link to PD: is time relevant?

The circadian clock is an internal timekeeping system that orchestrates the daily rhythms of physiological processes in nearly all living organisms. In mammals, this clock is regulated by a central pacemaker located in the suprachiasmatic nucleus (SCN) of the hypothalamus, which is synchronized via environmental timing cues, known as “Zeitgebers”, the strongest being light for humans (see Box 2). The light is detected by a specialized set of neurons named intrinsically photosensitive retinal ganglion cells (ipRGCs) which are then transmitted to the SCN via the retinohypothalamic tract (see Box 2). The SCN, in turn, coordinates the body’s peripheral oscillators—found in virtually all tissues and cells—through intricate networks of neuronal and hormonal signals, ensuring synchronization of various physiological processes across the body^20^. The circadian clock not only governs regulation of sleep-wake cycles, but also influences metabolism, hormone release, and even gene expression. Disruptions to this finely tuned system have been linked to various health disorders, including metabolic diseases, mood disorders, and neurodegenerative conditions like PD, underscoring the critical role of circadian rhythms in maintaining overall health^21^.

**Fig. 1:**
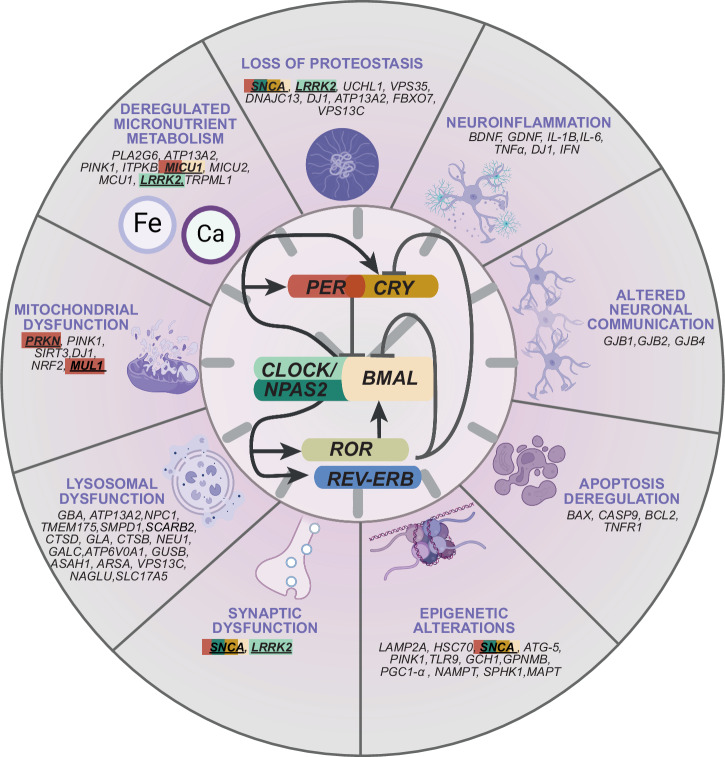
Cellular hallmarks of PD and connections to mammalian circadian clock. The hallmarks of PD can be categorized as loss of proteostasis, neuroinflammation, altered neuronal communication, apoptosis deregulation, epigenetic alterations, synaptic, lysosomal and mitochondrial dysfunctions, and deregulated micronutrient metabolism. The circadian clock is composed of self-sustained TTFL which include positive elements CLOCK and NPAS2 (a CLOCK paralog), BMAL, RORs, and negative elements PERs, CRYs, and REV-ERBs that drive ~24-h rhythmic expression of various target genes, both at the mRNA and protein levels. The core-clock genes regulate the expression of several other genes involved in hallmarks of PD highlighted in the figure with rectangles in corresponding colors for each core-clock gene and the genes connected to more than one core-clock gene are multi-colored. If the evidence was not explicitly found in the reported PD studies, the corresponding genes are left unmarked. Created with BioRender.com.

Cellular clocks function as molecular oscillators, driven by transcriptional and translational feedback loops (TTFLs) composed of transcriptional activators and repressors (Fig. 1). In humans, each circadian cycle begins with the dimerization of positive regulators, CLOCK (or it’s paralog NPAS2) and BMAL1 (also known as ARNTL), which bind to enhancer sequences, such as E-boxes, in the promoter regions of the negative regulators *PER*(*PER1,2,3*) and *CRY* (*CRY1,2*), initiating their expression^22^. The *Retinoid-related Orphan Receptors* (*RORs*) and *nuclear receptor subfamily 1 group D* (*NR1D, also known as REV-ERBs*) form an interconnected feedback loop that competes for ROR binding element (RRE) sites on *BMAL1*, fine-tuning the oscillation’s robustness. The accumulated PER and CRY proteins form a complex that inhibits BMAL1/CLOCK-mediated transcription at dawn, effectively ending one circadian cycle and a new cycle begins once these complexes are degraded. Post-translational modifications, such as the phosphorylation of PER by the *casein kinase 1* (*CK1*) gene family, further fine-tune the timing of circadian activity^23^. Together this network generates oscillation with an ~24-h period, hence termed circadian (Latin *circa* and dies, meaning about a day)^24^. In addition to the period, other important parameters characterize the circadian oscillation and can be quantified are: mesor (the adjusted mean around which the rhythm oscillates), amplitude (the deviation from mesor measured from peak or trough of the rhythm), and phase (the timing of the rhythm’s peak) (see also Box 2).

The core-clock TTFLs regulate further the circadian expression of clock-controlled genes (CCGs) by acting on E-boxes, D-boxes, RREs, or cAMP response elements (CREs). These CCGs govern a wide range of cellular functions, including cell cycle regulation, immune responses, and metabolism^25–27^. It is estimated that between 20 and 50% of gene expression in mammals, including humans, exhibits circadian rhythmicity in at least one tissue, highlighting the pervasive influence of circadian clocks on physiological processes^28–30^.

Disruption of the circadian system has been increasingly implicated in PD that may in part be explained with the degeneration in brain regions that regulate sleep and autonomic functions^31–35^. Clinically, these brain changes may manifest with disrupted thermoregulation and abnormal blood pressure patterns^36,37^. Postmortem studies revealed α-synuclein buildup and significant volume loss in the brainstem, particularly in the dorsal motor nucleus of the vagus, the region associated with gastrointestinal-autonomic regulation and the pontomesencephalic tegmentum, a region which consist of cholinergic, GABAergic, and glutamatergic neurons, found also linked to rapid eye movement sleep behavior disorder (RBD) symptoms^38–40^. RBD causes abnormal behavior during the REM sleep stage and is recognized as a prodromal stage for PD^41^, affecting up to 80% of PD patients in later course of the disease^42^. In addition, atrophy of the SCN previously observed in histology experiments^43^, which may explain the weakened body temperature and altered melatonin rhythms under the direct SCN control. Altogether, these underscore neurodegeneration in specific brain regions may contribute to the circadian alterations observed in PD patients. However, the precise role of circadian dysfunction in the onset and progression of these symptoms remains further to be elucidated.

The majority of PD patients, particularly those in the advanced stages of the disease, experience significant sleep disturbances as previously mentioned^44,45^. Earlier studies using wrist-worn actigraphy have documented substantial alterations in the sleep-wake cycles of PD patients, characterized by reduced amplitude and increased nocturnal activity^46,47^ (see Box 2). Another study confirmed that both early-stage PD patients (Höhn and Yahr (H&Y) stage I–II, *N* = 95) and late-stage PD patients (H&Y stage III-IV, *N* = 62) exhibit a reduction in the amplitude of their circadian rhythms compared to controls (*N* = 1111)^48^ (Table 1). Notably, phase advancement was observed exclusively in the late-stage group.

**Table 1:** Summary of studies analysing circadian rhythms in Parkinson’s disease context

Study	Species	Effects on circadian rhythm	Reference
PD patients with and without depression	Human	Altered circadian rhythm of core body temperature in PD patients with depression	Suzuki et al., 2007^59^
Total leukocytes of PD patients	Human	Altered peripheral molecular clock and BMAL1 levels correlate positively with PD severity	Cai et al., 2010^68^
Total leukocytes of PD patients	Human	Decreased expression of *BMAL2* in PD patients	Ding et al., 2011^69^
Twenty-nine PD patients divided into unmedicated and medicated groups	Human	The regulation of circadian phase and sleep timing as well as melatonin secretion is affected by dopaminergic treatment	Bolitho et al., 2014^58^
Idiopathic PD (IPD) patients with H&Y stage II-IV, assuming a stable dose of PD medications for at least 4 weeks before the study screening and during the entire study period	Human	Circadian dysfunction may be the cause of excessive sleepiness in PD	Videnovic et al., 2014^57^
Patients with early-stage Parkinson’s disease	Human	Sleep dysfunction in early Parkinson’s disease as a consequence of altered circadian rhythm	Breen et al., 2014^55^
Chinese Han PD patients	Human	Significant association between genetic polymorphisms in *ARNTL* and *PER1* clock genes and sporadic PD in a Chinese population	Gu et al., 2015^60^
Idiopathic PD (IPD) patients with H&Y stage I-III	Human	Association between circadian rest-activity rhythm and cognitive function in PD	Wu et al., 2018^52^
PD patients with and without cardiovascular dysautonomia	Human	A study confirming the alterations in circadian blood pressure rhythm in PD	Milazzo et al., 2018^34^
Chinese patients with PD	Human	Correlation between *CLOCK* 3111T/C gene variant and motor fluctuation and sleep disorder in Parkinson’s disease	Lou et al., 2018^62^
In vitro and in vivo 6-OHDA induced models of PD	Human (SH-SY5Y cells) and rat	Dysfunctional circadian clock alters antioxidative response in PD	Wang et al., 2018^66^
Fibroblasts from genetic PD patients carrying parkin mutations	Human	Link between mitochondrial alterations and deregulation of the molecular clockwork in Parkin mutated human fibroblasts	Pacelli et al., 2019^65^
Idiopathic PD (IPD) patients with H&Y stage I-III	Human	Rest-activity disruption in PD patients, associated with motor symptom severity and H&Y Stage.	Brooks et al., 2020^49^
Dopaminergic-treated Japanese PD patients	Human	BLT improves sleep causing a circadian phase shift	Endo et al., 2020^19^
PD patients with H&Y stage I-III	Human	Increased levels of *BMAL1* following melatonin administration	Delgado-Lara et al., 2020^108^
Old men without PD at baseline	Human	Decreased circadian amplitude, mesor, or robustness linked to increased risk of Parkinson’s disease	Leng et al., 2020^51^
Early-stage (Hoehn-Yahr I and II) and late-stage (Hoehn-Yahr III–V) PD patients	Human	Significant differences in the circadian activity rhythm parameters in PD patients in comparison to the controls	Obayashi et al., 2021^48^
Peripheral blood mononuclear cells (PBMCs) of PD patients	Human	Altered expression of peripheral clock genes and circulating melatonin levels in PD patients	Li et al., 2021^67^
PD patients and human colorectal carcinoma cell line HCT116 with genetically altered circadian clock	Human	Common differentially expressed genes between IPD patients and circadian clock mutated cells	Yalcin et al., 2021^70^
In vitro 6-OHDA induced model of PD	Human (SH-SY5Y cells)	Disruption of the circadian clock genes	Su et al., 2023^143^
Patients with mild to moderate PD	Human	BLT based on the patient’s personal chronotype improves sleep disturbances and motor symptoms in PD	Feigl et al., 2024^132^
Alpha-synuclein overexpressing (ASO) mice	Mouse	Circadian abnormalities, with reduced neuronal firing rates in the SCN and disrupted sleep/activity cycles	Kudo et al., 2011^144^
MitoPark mouse	Mouse	Impaired circadian control of rest/activity rhythms as a consequence of the loss of the midbrain DA neurons	Fifel and Cooper, 2014^145^
6-OHDA mouse model of PD	Mouse	Genetic abrogation of the circadian nuclear receptor REV-ERBα increases the vulnerability of DA neurons to neurotoxic insults	Kim et al., 2018^146^
MPTP-treated *Bmal1*^−/−^ mice	Mouse	Role of *BMAL1* in the survival of DA neurons and in microglia-mediated neuroinflammation	Liu et al., 2020^147^
MPTP-induced mouse model of PD	Mouse	Circadian clock protein Rev-erbα attenuates neuroinflammation in PD pathology	Kou et al., 2022^98^
6-OHDA mouse model of PD	Mouse	*REV-ERB*α is a potential therapeutic target for mood disorders linked to circadian disturbances in PD	Kim et al., 2022^100^
6-OHDA rat model of PD	Rat	Disrupted circadian behaviors and altered circadian expression of *Per2*	Ben and Bruguerolle, 2000^95^
6-OHDA rat model of PD	Rat	Dopamine system regulates circadian activity	Gravotta et al., 2011^94^
Drosophila models of PD with mutations in MUL1 and PARKIN gene	Drosophila	Disruption of the circadian clock and of the circadian rhythms in behavior as a consequence of mul1 and parkin mutations	Doktór et al., 2019^79^

In a retrospective observational clinical study involving 13 idiopathic PD (IPD) patients (Box 1) with H&Y stage I-III demonstrated the potential of actigraphy in clinical staging, showing that differences in activity patterns across PD stages could serve as biomarkers, pending validation in larger cohorts^49^. Another study which analysed 7-day actigraphy data from 88 patients with idiopathic REM sleep behavior disorder (iRBD), alongside 44 non-RBD controls and 44 clinically diagnosed synucleinopathy patients, revealed significant disruptions in rest-activity patterns in iRBD patients^50^. These disruptions included increased daytime napping, fragmented sleep, and lower overall physical activity levels^50^. Monitoring these patterns could be crucial in predicting the progression of synucleinopathy, potentially facilitating earlier disease detection.

Furthermore, a study tracking 2930 men without a PD diagnosis at baseline over an 11-year follow-up period found that individuals at high risk for PD exhibited reduced amplitude, mesor and less robust sleep-wake cycles^51^. Stable rest-activity cycles have also been correlated with better cognitive performance, as demonstrated in a study of 35 PD patients^52^. Interestingly, these cognitive benefits were linked specifically to rest-activity stability, rather than sleep efficiency^52^, suggesting that endogenous circadian regulation, rather than homeostatic sleep regulation, plays a pivotal role in mediating cognitive abilities in PD.

Circadian disruptions in PD significantly impact hormonal secretion, including cortisol and melatonin, as well as core body temperature and blood pressure rhythms, all of which are directly regulated by the circadian clock^53,54^. In a study involving 30 PD patients and 15 controls, plasma cortisol levels were found to be elevated in PD patients, whereas circulating melatonin was reduced despite no observable change in the phase of the rhythms^55^. A recent systematic review investigating cortisol alterations in PD identified out of 21 studies assessed ten studies reported disrupted cortisol rhythms, with the majority (seven out of ten) indicating elevated cortisol levels^56^. However, some studies failed to find significant changes or even observed opposite trends, highlighting the need for further research to validate these findings. Conversely, plasma melatonin levels have been shown to be significantly lower in PD patients compared to controls, as measured by radioimmunoassay in a study with 20 PD patients and 15 controls^57^. Another study, which included PD patients undergoing medical treatment (*N* = 16), unmedicated patients (*N* = 13), and healthy controls (*N* = 27), revealed that medicated patients exhibited a longer phase angle of entrainment (see Box 2 for definition) indicating a larger time difference between phase of the circadian rhythm and the external cue, calculated by subtracting salivary dim light melatonin onset (DLMO) from habitual sleep onset^58^. In addition, higher melatonin levels were observed compared to the non-medicated group^58^. These findings suggest that alterations in melatonin rhythms could be influenced by dopaminergic treatments (Box 1).

While overall circadian patterns in core body temperature are generally preserved in PD, the mesor of core body temperature is lower in PD patients, particularly in those with co-existing depression^54,59^. Additionally, more than 50% of PD patients exhibit abnormal blood pressure rhythms, including a phenomenon known as “reverse dipping”, where nighttime blood pressure is higher than daytime levels^34^.

Recent advances in high-throughput sequencing and genomic analysis have significantly expanded our understanding of the molecular mechanisms underlying PD. In a genotyping study involving 1394 PD patients and 1342 controls, specific single nucleotide polymorphisms (SNPs) in circadian clock genes were found to be associated with distinct clinical manifestations of PD^60^. For instance, the *ARNTL* rs900147 variant was significantly associated with tremor-dominant PD (see also Box 1), while the *PER1* rs2225380 variant correlated with postural instability and gait difficulty-dominant PD (Box 1), suggesting that genetic variations in circadian clock genes may influence the clinical presentation of the disease.

Further supporting this notion, a study involving 646 PD patients and 352 controls identified the *CLOCK* gene rs1801260 polymorphism as being associated with a twofold increased risk of developing PD^61^. In the same population, the *CLOCK-3111T/C* variant was linked to motor fluctuations and sleep disorders^62^. These findings indicate that alterations in clock genes may impact mitochondrial bioenergetics, autophagy, and neuroendocrine function, thereby contributing to PD pathogenesis^63^. For example, mutations in the parkin RBR E3 ubiquitin protein ligase (*PRKN)* gene have been correlated with the circadian regulation of mitochondrial function^64,65^, while alterations in the antioxidative NAD-dependent deacetylase sirtuin-1 (*SIRT1)* gene have been shown to affect circadian rhythms and oxidative stress regulation, contributing to neurodegeneration^66^.

In addition to polymorphisms, changes in clock gene expression profiles have been observed in PD patients. Decreased *BMAL1, CLOCK, CRY1, PER1, and PER2* expression reported previously in the peripheral blood of PD patients (*N* = 326) compared to controls (*N* = 314)^67^. Reduction in *BMAL1* expression was further confirmed in whole-blood samples (*N* = 17 PD patients, *N* = 16 controls)^68^ where a downregulation of *BMAL2* was later reported in the same cohort^69^ (Table 1). Additionally, Breen et al. documented disrupted circadian rhythms in PD patients, characterized by abolished *BMAL1* rhythms and increased expression of *PER2* and *REV-ERBα* (at 4 AM), in a group of 30 PD, and 15 controls^55^. Interestingly, nocturnal *BMAL1* expression was found to correlate with PD symptom severity, suggesting its potential as a predictive marker for disease progression^68^.

Moreover, a recent study assessing peripheral clock-gene expression in hair samples from 17 PD patients undergoing dopaminergic therapy found that those who responded positively to evening bright light therapy (BLT) exhibited a phase shift in *PER3* expression^19^ (see Box 2 for detailed definitions). This phase shift appeared to impede the restoration of circadian rhythms, potentially explaining improvements in sleep disturbances observed in these patients^19^. Our group has also contributed to this growing body of evidence by conducting a transcriptomics analysis of the circadian clock network, revealing weaker correlations in clock gene expression in PD patients (*N* = 205) compared to age- and sex-matched controls (*N* = 233), indicative of disrupted circadian regulatory mechanisms^70^. Collectively, these findings underscore the significant impact of circadian disruptions in PD. Understanding these disruptions offers a promising avenue for developing targeted interventions aimed at restoring circadian function, ultimately enhancing the management of PD.

Box 2 Glossary of key terms related to circadian rhythms

**Table:** 

**Term**	**Definition**
**Actigraphy**	A non-invasive method used for assessment of sleep and activity patterns over time using a wearable device with sensors, typically an accelerometer.
**Amplitude**	Defined by the maximum (peak) or minimum (trough) point of an oscillation divided by the rhythm adjusted mean (see also definition of mesor). For a symmetrical wave, the amplitude is equal to half of the range of oscillation (peak-to-trough distance).
**Bright light therapy**	Exposure to a light source with a light density greater than 1000 lux to reset circadian rhythms.
**Chronobiology**	The field of research focusing on the interaction of time with biological systems.
**Circadian rhythm**	A rhythm with an approximately 24-h period that exists in the absence of input from any external cues meaning endogenous; can be reset by environmental factors such as light and maintains a stable period despite physiological temperature fluctuations known as temperature compensation.
**Cortisol**	The steroid hormone released by the adrenal gland that mediates metabolism, blood pressure, glucose levels, immune, and stress response. Cortisol has a robust circadian rhythm with a typical peak activity in the morning to promote wakefulness and reaches to its minimum at night.
**Dawn**	The morning time when daylight starts.
**Diurnal**	The activity or event that take place during the day, corresponding to the time from dawn until dusk.
**Dusk**	The evening time when daylight disappears.
**Entrainment**	The synchronization of period of two oscillators. In context of circadian rhythms, the period of the internal clock is synchronized to the external system such as the solar day.
**Intrinsically photosensitive retinal ganglion cells**	A subset of photoreceptors located in the retina, which are activated by the photopigment melanopsin and mediate nonvisual effects of light.
**Melatonin**	A circadian expressed hormone secreted by the pineal gland that mediates sleep and wake timing. Melatonin secretion increases following onset of darkness and reaches to peak at night.
**Mesor**	The rhythm adjusted mean around which the fitted cosine wave oscillates.
**Nocturnal**	The activity or event that take place during the night, corresponding to the time from dusk until dawn.
**Period**	The duration of time after which a phase of the oscillation repeats, in case for circadian rhythms corresponds to ~24 h.
**Phase**	The time point when the maximum activity or expression of a rhythm occurs. Primarily, referred to the phase angle corresponding to the peak of a cosine wave which is fitted to the raw time-series data.
**Phase angle of entrainment**	It refers to the relationship between the timing of the circadian clock and an external timing cue. A longer phase angle of entrainment indicates a larger time difference between phase of the circadian rhythm and the external cue.
**Phase shift**	The change along the time axis of an oscillation advancing or delaying it.
**Polysomnogramy**	A systematic sleep medicine approach to record multiple physiological parameters such as brain waves, eye movements, muscle activity, heart rate, and breathing patterns to identify the causes of sleep disturbances.
**Retinohypothalamic tract**	The neuronal pathway that transmits light input to the circadian pacemaker suprachiasmatic nucleus.
**Zeitgeber**	An environmental cue that can entrain circadian rhythms, such as light, food, or social cues, literally meaning “time giver“ from German.

## Molecular mechanisms of PD

At the molecular level, the involvement of several complex and overlapping mechanisms complicates our understanding of the etiology of PD. Several of these processes have also been found to be linked to the circadian clock, including loss of proteostasis, disruption in micronutrient metabolism, synaptic dysfunction, and mitochondrial and epigenetic alterations (Table 2 and Fig. 1).

**Table 2:** Compilation of research articles showing a correlation between PD and core clock genes

PD genes	Clock genes	PD-clock genes correlation	Reference
*SNCA* *SNCA* *SNCA*	*CRY* *NPAS2* *PER*	Decreased *CRY2* gene expression in a *SNCA* overexpressing mouse model. Decreased *NPAS2* gene expression in a *SNCA* overexpressing mouse model. Decreased *PER* gene expression in a *SNCA* overexpressing mouse model.	Hentrich et al., 2018^73^ Hentrich et al., 2018^73^ Hentrich et al., 2018^73^
*SNCA*	*BMAL*	Downregulation of *BMAL* in SNCA^A53T^ mice and in PC12 cells overexpressing *SNCA*.	Liu et al., 2023^148^
*LRRK2*	*CLOCK*	Decreased levels of CLOCK protein in *Lrrk2*^G2019S^ transgenic mice following chronic sleep deprivation.	Liu et al., 2022^74^
*MICU1*	*BMAL*	Higher expression of *BMAL* and *MICU1* genes in cardiac mitochondria during sleep period.	Abdel-Rahman et al., 2021^149^
*MICU1*	*PER*	Lower expression of *PER2* gene and higher expression of *MICU1* gene in cardiac mitochondria during sleep period.	Abdel-Rahman et al., 2021^149^
*MUL1* *PARK*	*PER* *PER*	Difference in the daily expression profile of clock genes and PER protein in a mutated *mul1* Drosophila model of PD. Difference in the daily expression profile of clock genes and PER protein in a mutated *park* Drosophila model of PD.	Doktór et al., 2019^79^ Doktór et al., 2019^79^

Ageing is a major factor for the development of PD, and is associated with a decline in the activity of the proteostasis network, possibly leading to increased aggregation and accumulation of aSyn, which is thought to impact on the function and viability of dopaminergic neurons^71^ (Box 1). However, it is still unclear whether such effects are due to a gain of toxic function due to aggregation, or due to a depletion of the normal function of aSyn, which is thought to be related to the trafficking and fusion of synaptic vesicles with the plasma membrane. Altered proteostasis is also responsible for synaptic dysfunction, which is thought to be an early event in PD^72^. The link between alterations in the circadian clock and loss of proteostasis has been suggested by the decreased expression of the clock genes *CRY*, *NPAS2,* and *PER* in a PD mouse model overexpressing human *SNCA* that shows age-related alterations in the hippocampal transcriptome^73^. Another study reported decreased levels of the CLOCK protein in transgenic mice carrying a mutation in the *LRRK2* gene, which is strongly implicated in familial and sporadic forms of PD^74^. aSyn aggregation may also induce cell senescence, causing cell cycle arrest and triggering a powerful inflammatory response, thereby contributing to PD progression^75^. In fact, altered levels of cellular senescence markers have been found in the SNc (Box 1) of postmortem human PD brain tissue^75^. Consistently, senescent glial cells were shown to worsen the pathogenesis of PD by inducing chronic neuroinflammatory processes and by reducing aSyn clearance due to reduced autophagic activity^76^.

Another major molecular alteration in PD is mitochondrial dysfunction, associated with selective dopaminergic neurodegeneration and ROS production (Fig. 1). In particular, inhibition of mitochondrial complex I by rotenone or MPTP (1-methyl-4-phenyl-1,2,3,6-tetrahydropyridine), induces parkinsonism in humans and was shown to induce aSyn aggregation in various model systems^77^. Mitochondria are major producers of reactive oxygen species (ROS) in the cell and, therefore, can stimulate inflammatory responses through the release of their constituents and metabolites into the cytosol or in the extracellular environment^78^. The correlation between CR dysfunction and mitochondrial alterations has been demonstrated in fibroblasts from PD patients carrying Parkin mutations, which show an altered bioenergetic rhythmicity related to the deregulation of clock genes such as *PER2*^65^. Moreover, in Drosophila models of PD, mutations in Parkin and in mitochondrial ligase (*MUL1*) affect CR of behavior and, in general, the molecular mechanism of the circadian clock^79^ .

PD genetics has also provided important insight into the molecular underpinnings of the disease and have implicated pathways such as the ubiquitin proteasome system, autophagy, or vesicular transport^71,80^. Genomic instability due to ageing, leads to nuclear DNA damage, and this has been described in several studies in PD^81,82^. Moreover, DNA double- (DSBs) or single-strand breaks (SSBs) accumulate in the brains (and particularly in the midbrain) of patients with PD^83^. Altered gene expression may also derive from a different epigenetic regulation which modifies gene expression without altering their sequence^84^. The best-known epigenetic modification is DNA methylation, entailing the addition of a methyl group to a particular nucleotide (cytosine) in CpG islands. In PD, several laboratory studies have started to investigate the epigenetic landscape, but it is still unclear how this contributes to disease.

Other epigenetic modifications like those affecting chromatin remodeling (e.g., the different histone modifications)^85^ or, at RNA level, those regulated by non-coding RNAs^86^, or by epitranscriptomic modifications which affect translation by post-transcriptional chemical modifications of RNA molecules^87^, may also play an important role in PD (Fig. 1). Among the latter, the most abundant modification is RNA-methylation at the N6-position of adenosine (m6A), which plays an important role in the epitranscriptomic regulation of pathways associated with PD, such as those related to motor function^88^, or those related to regulating the death of dopaminergic neurons^89^(Box 1). Interestingly, in PD brains, a significant reduction in the abundance of m6A-modified RNAs has been observed in three regions (frontal and cingulate gyrus cortices and hippocampus)^90^ (Box 1). Some studies have investigated the correlation between clock genes and PD at the epigenetic level. Methylated promoters of the clock genes *CRY* and *NPAS2* have been found in patients with PD^91^, and a high frequency of methylation in the CpG islands of circadian genes has been seen in patients with Dementia with Lewy bodies^92,93^.

Additional studies will be necessary in order to determine the complex molecular mechanisms underlying PD, and to develop innovative and effective therapeutic strategies.

## Circadian rhythms—impact on disease management

Exploring the impact of CR disruption in PD opens new avenues for therapeutic interventions ranging from pharmacological to non-pharmacological applications to restore circadian dynamics or using the clock profiles as biomarkers for disease monitoring and management. Animal models are indispensable for advancing our understanding of circadian disruptions in PD, particularly given the challenges associated with accessing brain tissue in human subjects. In Drosophila PD models, mutations in key genes like *mitochondrial ubiquitin ligase 1* (*mul1*), that regulates mitochondrial integrity and fusion–fission processes, and *parkin* (*park*), which facilitates the ubiquitination of mitochondrial substrates, have been shown to prolong activity rhythms and alter core-clock machinery^79^. Specifically, *park1*^1^ mutants exhibited a phase delay of ~3 h in the rhythmic expression of *per* and *tim* genes, while the protein-level rhythmic activity of PER was completely abolished in both *park1*^*1*^ and *mul1*^A6^ mutants^79^ (Table 1). Similarly, in rat models, disrupted circadian behaviors, physiological outputs, and altered circadian expression of *Per2* have been observed^94,95^.

In *Bmal1* knockout mice injected with MPTP, a compound that selectively depletes dopaminergic neurons in the substantia nigra (Box 1), circadian dysregulation was associated with a significant reduction in dopaminergic neurons and transmitters, as well as altered inflammatory and antioxidative defense responses, as indicated by increased microglial and astrocyte activity^96^. Another core-clock component, *REV-ERBα* influences energy metabolism and was found to protect against neuroinflammation in the MPTP-induced-mouse model of parkinsonism^97,98^. Moreover, it inhibits the expression of the rate-limiting enzyme, tyrosine hydroxylase, required for dopamine biosynthesis thereby ensures circadian activity of dopaminergic neurons (Box 1) and regulates mood^99^. In the 6-hydroxydopamine (6-OHDA) mouse model of parkinsonism, animals exhibited depression and anxiety symptoms, similar to human sundowning syndrome^100^. Administering the *Rev-Erbα* antagonist SR8278 rescued these behaviors in a time-dependent manner, effective only at subjective dawn and not at dusk^100^ suggesting that the restoration of circadian rhythms may help with neuropsychiatric symptoms in PD.

Currently we lack curative therapies for PD. Among the symptomatic treatment options, levodopa (L-3,4-Dioxyphenylalanine, L-DOPA), a precursor to dopamine, is the most widely used (Box 1)^101^. L-DOPA is used as a replacement for the reduced levels of dopamine in the brain, with the aim to counteract the bradykinetic symptoms that are typical of PD. It is used in combination with peripheral decarboxylase inhibitors such as carbidopa or benserazide, which prevent its premature metabolization to dopamine by the enzyme aromatic L-amino acid decarboxylase (AADC), before reaching the brain^102^. Interestingly, these dopaminergic treatments affect CR of PD patients by phasing forward, for example, the melatonin rhythm^103^.

In humans, melatonin is one of the best studied compounds in the context of restoring circadian dynamics, which has been shown to enhance subjective sleep quality in PD and to exhibit antioxidative properties^104,105^. A slight improvement in nocturnal sleep in PD patients taking 50 mg of melatonin (in comparison to 5 mg placebo) has been observed, though the improvement was short-term (roughly 10 min)^106^. Medeiros and colleagues on the other hand observed improved subjective sleep quality in PD patients taking a much lower dose (3 mg/day) of melatonin for a month, despite no significant change was detected in polysomnography (PSG) results^107^. Delgado-Lara et al. reported increased *BMAL1* gene expression in PD patients who were administered 25 mg of melatonin for 3 months, particularly in the morning, suggesting the improvement in PD symptom management is linked to restoration of core-clock machinery^108^. In a recent systematic review where seven randomized controlled trials were assessed, melatonin was suggested as a safe and well-tolerated compound for the management of insomnia in PD patients albeit there was no improvement in daytime sleepiness or RBD symptoms^109^. Other evidence showed a positive improvement of motor and nonmotor PD symptoms (including sleep disorders) in PD patients following treatment with cannabis^110^. With the development of new pharmacological compounds, such as small-molecule modulators, a restoration of disrupted CR has been explored. For example, compounds that inhibit casein kinases led to period-lengthening effects in human osteosarcoma cells (U2OS) and mouse embryonic fibroblasts (MEFs)^111^, proposed to be linked to neuroprotective actions of *CKI-δ* encoded by *CSNK1D*. *CKI* directly acts on core-clock post-translational modifications by phosphorylation of PER^23^ pending to be validated further in vivo. Despite these exciting developments, the exact molecular mechanisms behind clock disruption and PD are yet to be further elucidated. Moreover, clinical studies that take individual circadian rhythms into account are scarce, and further research in this field is timely.

**Fig. 2:**
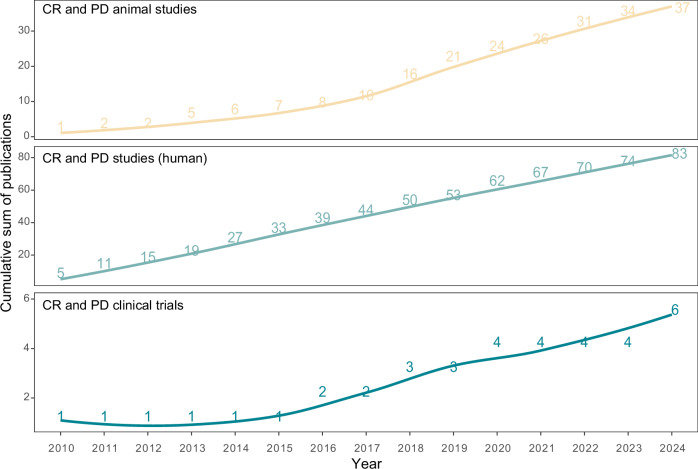
Emerging role of the circadian clock in PD and its translation to clinical settings. Number of PubMed publications since 2010 until end of 2024 considering CR. Studies were categorized based on research type: studies with animal models (beige), only in humans including in vitro evidence and non-interventional clinical studies such as observational studies (light blue) and clinical trials (dark blue). Trend charts represent cumulative summary of papers over the years, locally estimated scatterplot smoothing (LOESS) regression used to depict trend lines.

To demonstrate the gap between basic research studies versus clinical studies on PD and the circadian clock, we carried out a PubMed search covering publications starting from 01.01.2010 until 31.12.2024. We used MeSH terms and keywords related to “circadian clock” and “Parkinson’s Disease” (see Box 3 for the detailed search strategy). We initially focused on original research articles, based on only animal models or human studies (including in itro evidence and non-interventional clinical studies such as observational studies), excluding review papers, commentaries or editorial publications, clinical trials and study protocols. For animal models we used a previously published search strategy^112^ (see also Box 3). Next, we included only clinical trials to determine the number of publications where “circadian clock” was used as a direct target for an intervention. We selected publications in English language for further analysis. Since this is not a systematic review, it was beyond the scope of the current review to carry out manual curation regarding title and abstract review. Nevertheless, our search underscored despite the growing interest in the circadian field and its connection to pathologies; there is a notable shortage of mechanistic insights in both animal models and human studies (*N*_total publications_ = 37 with animal models and 83 human studies without a clinical intervention endpoint until the end of 2024, respectively), as evidenced by the low number of studies overall (Fig. 2).

The number of clinical trials in this overlap has remained consistently scarce (*N*_total publications_ = 6) (Fig. 2). The persistent scarcity of clinical trials in circadian research is due to need for time-series data, rather than a snapshot at a certain time-of-day, making them more costly and complex to conduct in clinical settings. For assessment of CR peripheral markers (e.g., hormonal levels such as cortisol, melatonin or core body temperature) are widely used to estimate the SCN phase due to its inaccessibility in humans, though they require time-series collection for accuracy^113,114^. DLMO assessment is considered as gold-standard in the field, but DLMO values still govern inherent variability, and the procedure requires an overnight clinical stay, making it impractical for routine use. Emerging methods include high-dimensional assessments (e.g., DNA/transcriptome/metabolome profiling) or from sampling of physiological and environmental variables. Actigraphy and wearable devices (e.g., FitBit watches, Oura ring) are widespread used in this context which allow continuous monitoring of CR outputs (e.g., body temperature, heart rate, activity), but they do not capture underlying circadian gene and protein expression changes. To overcome this, several circadian gene expression assessment methods have been developed in recent years by our group and others^115–118^. Using a non-invasive approach TimeTeller^®^ has been used to characterize molecular clock profiles from saliva samples^115,119^. In an ongoing non-interventional, observational study, we planned to recruit 70 PD patients and 20 controls, to characterize the circadian profiles and identify the changes between the groups^120^. While in vitro diagnostic (IVD) methodologies like TimeTeller^®^ offer a non-invasive, at-home solution using saliva samples, there is still no widely accepted common molecular tool to model CR based on core-clock gene expression, which is essential for precision medicine. This gap is particularly crucial in PD, where there is a lack of definitive diagnostic criteria and underscores the need for the development of new biomarkers to enhance early detection, accurate diagnosis, and effective monitoring of disease progression. Exploring CR alterations in this link holds potential essential not only for developing disease biomarkers but also for improving symptom management, understanding disease heterogeneity, and monitoring disease progression.

To complement pharmacological applications, the potential benefits of various non-pharmacological interventions have been investigated. In this context, BLT, which acts on retinal inputs to the circadian system, demonstrated improvements in sleep quality, insomnia, daytime sleepiness, depression, and motor symptoms in PD patients^121–126^. Moreover, this therapy also counteracts the negative effects of dopamine treatment on sleep by modulating circadian rhythms^19^. BLT is believed to work by improving monoaminergic function and inhibiting circadian melatonin secretion, following exposure of the retina to light^127–131^. This treatment has recently received a major boost with the development of standardized and customized protocols based on the patient’s personal chronotype^132^. In an early, pilot randomized placebo-controlled double-blind study with 36 PD patients who received BLT (see Box 2) in the morning for two weeks (30 min per day, 7500 Lux for treated group, whereas 950 lux for placebo) significant improvements in tremor and depression symptoms were reported^129^. In another pioneering study, 12 IPD patients with nonmotor symptoms received BLT before bedtime (between 1000 and 1500 Lux for 60–90 min) over 2 to 5 weeks period, resulting in improvements in sleep onset and fragmentation, mood, and motor symptoms specifically for bradykinesia (Box 3) and rigidity^124^. In a follow-up retro-perspective open-label study 129 PD patients under dopaminergic treatment were analysed and improvements in sleep, mood and motor symptoms were confirmed under similar BLT conditions but only for compliant patients, emphasizing the importance of continued light exposure for sustained benefits^125^. Another clinical trial examined 31 PD patients receiving dopaminergic treatment, comparing BLT to dim-red light therapy over two weeks (1 h of BLT with 10,000 lux or dim-red LT with less than 300 lux between 09:00–11:00 and 17:00-19:00)^122^. Patients exposed to BLT showed enhanced sleep quality metrics, including reduced sleep fragmentation and daytime sleepiness, highlighting BLT’s potential in managing PD-related sleep disturbances^122^. In a recent retrospective open-label longitudinal study investigating the long-term impact of BLT administered before bedtime (1 h per day at 3000–4000 Lux for a period of 2 to 5 years) in 140 PD patients, ongoing improvements in insomnia, sleep quality, and nocturnal movement were observed^121^. Further studies are needed though to optimize the timing, duration, and parameters of light therapy for effective management of PD.

Physical exercise, which is bidirectionally influenced by circadian rhythms, plays a critical role in PD. Regular exercise can significantly improve motor functions, alleviate symptoms such as rigidity and bradykinesia (Box 1), and improve the overall quality of life for PD patients^133^. High-intensity exercise acts in this regard, with significant improvements on the sleep quality of PD patients^134^. Furthermore, in combination with an overnight and morning fast, exercise in the morning may slow the progression of PD by acting in cooperation with circadian rhythms to counteract mitochondrial dysfunctions, implicated in the pathogenesis of the disease^135^.

Research in both animal models and humans suggests there might be an optimal time to maximize exercise benefits^136^. Circadian variations in core body temperature, hormone levels, and muscle function affect exercise performance throughout the day^137^. In addition, circadian variation in gene expression also influences athletic performance^136,138^. In our recent study, which analysed the circadian profiles of core-clock genes among 15 healthy, physically active participants who performed physical activities at different times of the day, we identified *PER2* peak timing as a key predictor for the timing of exercise performance^115^. Despite these significant advancements determining the best exercise timing remains complex due to various factors impacting exercise outcomes, including the type, intensity, duration, and frequency of exercise, impact on underlying metabolic circuits and specific symptoms being targeted^139,140^. Underlying circadian disruptions unique to PD patients might further influence these outcomes. Activity trackers offer valuable insights in this context for the monitoring. A previous study highlighted that the step count is particularly useful to accurately assess daily variations in physical activity for PD patients after two days of data collection and can be used to optimize exercise prescriptions^141^. Moreover, a recent exploratory study using a hip-worn accelerometer found that individuals with PD who experience pain are notably less active (<4200 steps per day), depicting reduced activity levels especially in the morning hours^142^. Understanding such variations in CR may be used to personalize exercise schedules to maximize benefits for PD patients.

Box 3 Summary of PubMed search strategies Publications were categorized into four groups—PD and CR animal studies; PD and CR human studies (excluding clinical trials); PD and CR clinical trials, and PD clinical trials without consideration of CR. Reviews were excluded, and only publications in English from 2010 until the end of 2024 were included. Data were retrieved on 03.03.2025.

**Table:** 

**Theme**	**Search Strategy**
PD and CR animal studies	("Parkinson disease"[Mesh] OR "Idiopathic Parkinson's Disease"[tiab] OR "Parkinson's Disease, Lewy Body"[tiab] OR "Primary Parkinsonism"[tiab] OR "Paralysis Agitans"[tiab]) ("Circadian rhythm"[Mesh] OR "circadian rhythm"[tiab] OR "Circadian Clock"[tiab] OR "Clock, Circadian"[tiab] OR "Clocks, Circadian"[tiab] OR "Clocks, Circadian"[tiab] OR "Circadian Clock System*"[tiab] OR "Circadian System"[tiab] OR "Circadian Timing System*"[tiab] OR "Circadian Rhythm*"[tiab] OR "Twenty-Four Hour Rhythm"[tiab] OR "Twenty Four Hour Rhythm*"[tiab] OR "Nyctohemeral Rhythm*"[tiab] OR "Nycthemeral Rhythm*"[tiab] OR "Diurnal Rhythm"[tiab] OR "Rhythms, Diurnal"[tiab]) AND (("animal experimentation"[MeSH Terms] OR "models, animal"[MeSH Terms] OR "invertebrates"[MeSH Terms] OR "Animals"[Mesh:noexp] OR "animal population groups"[MeSH Terms] OR "chordata"[MeSH Terms:noexp] OR "chordata, nonvertebrate"[MeSH Terms] OR "vertebrates"[MeSH Terms:noexp] OR "amphibians"[MeSH Terms] OR "birds"[MeSH Terms] OR "fishes"[MeSH Terms] OR "reptiles"[MeSH Terms] OR "mammals"[MeSH Terms:noexp] OR "primates"[MeSH Terms:noexp] OR "artiodactyla"[MeSH Terms] OR "carnivora"[MeSH Terms] OR "cetacea"[MeSH Terms] OR "chiroptera"[MeSH Terms] OR "elephants"[MeSH Terms] OR "hyraxes"[MeSH Terms] OR “insectivora”[MeSH Terms] OR "lagomorpha"[MeSH Terms] OR "marsupialia"[MeSH Terms] OR "monotremata"[MeSH Terms] OR "perissodactyla"[MeSH Terms] OR "rodentia"[MeSH Terms] OR "scandentia"[MeSH Terms] OR "sirenia"[MeSH Terms] OR "xenarthra"[MeSH Terms] OR "haplorhini"[MeSH Terms:noexp] OR "strepsirhini"[MeSH Terms] OR "platyrrhini"[MeSH Terms] OR "tarsii"[MeSH Terms] OR "catarrhini"[MeSH Terms:noexp] OR "cercopithecidae"[MeSH Terms] OR "hylobatidae"[MeSH Terms] OR "hominidae"[MeSH Terms:noexp] OR "gorilla gorilla"[MeSH Terms] OR "pan paniscus"[MeSH Terms] OR "pan troglodytes"[MeSH Terms] OR "pongo pygmaeus"[MeSH Terms]) NOT ((animals[tiab] OR animal[tiab] OR mice[Tiab] OR mus[Tiab] OR mouse[Tiab] OR murine[Tiab] OR woodmouse[tiab] OR rats[Tiab] OR rat[Tiab] OR murinae[Tiab] OR muridae[Tiab] OR cottonrat[tiab] OR cottonrats[tiab] OR hamster[tiab] OR hamsters[tiab] OR cricetinae[tiab] OR rodentia[Tiab] OR rodent[Tiab] OR rodents[Tiab] OR pigs[Tiab] OR pig[Tiab] OR swine[tiab] OR swines[tiab] OR piglets[tiab] OR piglet[tiab] OR boar[tiab] OR boars[tiab] OR "sus scrofa"[tiab] OR ferrets[tiab] OR ferret[tiab] OR polecat[tiab] OR polecats[tiab] OR "mustela putorius"[tiab] OR "guinea pigs"[Tiab] OR "guinea pig"[Tiab] OR cavia[Tiab] OR callithrix[Tiab] OR marmoset[Tiab] OR marmosets[Tiab] OR cebuella[Tiab] OR hapale[Tiab] OR octodon[Tiab] OR chinchilla[Tiab] OR chinchillas[Tiab] OR gerbillinae[Tiab] OR gerbil[Tiab] OR gerbils[Tiab] OR jird[Tiab] OR jirds[Tiab] OR merione[Tiab] OR meriones[Tiab] OR rabbits[Tiab] OR rabbit[Tiab] OR hares[Tiab] OR hare[Tiab] OR diptera[Tiab] OR flies[Tiab] OR fly[Tiab] OR dipteral[Tiab] OR drosphila[Tiab] OR drosophilidae[Tiab] OR cats[Tiab] OR cat[Tiab] OR carus[Tiab] OR felis[Tiab] OR nematoda[Tiab] OR nematode[Tiab] OR nematoda[Tiab] OR nematode[Tiab] OR nematodes[Tiab] OR sipunculida[Tiab] OR dogs[Tiab] OR dog[Tiab] OR canine[Tiab] OR canines[Tiab] OR canis[Tiab] OR sheep[Tiab] OR sheeps[Tiab] OR mouflon[Tiab] OR mouflons[Tiab] OR ovis[Tiab] OR goats[Tiab] OR goat[Tiab] OR capra[Tiab] OR capras[Tiab] OR rupicapra[Tiab] OR chamois[Tiab] OR haplorhini[Tiab] OR monkey[Tiab] OR monkeys[Tiab] OR anthropoidea[Tiab] OR anthropoids[Tiab] OR saguinus[Tiab] OR tamarin[Tiab] OR tamarins[Tiab] OR leontopithecus[Tiab] OR hominidae[Tiab] OR ape[Tiab] OR apes[Tiab] OR pan[Tiab] OR paniscus[Tiab] OR "pan paniscus"[Tiab] OR bonobo[Tiab] OR bonobos[Tiab] OR troglodytes[Tiab] OR "pan troglodytes"[Tiab] OR gibbon[Tiab] OR gibbons[Tiab] OR siamang[Tiab] OR siamangs[Tiab] OR nomascus[Tiab] OR symphalangus[Tiab] OR chimpanzee[Tiab] OR chimpanzees[Tiab] OR prosimians[Tiab] OR "bush baby"[Tiab] OR prosimian[Tiab] OR bush babies[Tiab] OR galagos[Tiab] OR galago[Tiab] OR pongidae[Tiab] OR gorilla[Tiab] OR gorillas[Tiab] OR pongo[Tiab] OR pygmaeus[Tiab] OR "pongo pygmaeus"[Tiab] OR orangutans[Tiab] OR pygmaeus[Tiab] OR lemur[Tiab] OR lemurs[Tiab] OR lemuridae[Tiab] OR horse[Tiab] OR horses[Tiab] OR pongo[Tiab] OR equus[Tiab] OR cow[Tiab] OR calf[Tiab] OR bull[Tiab] OR chicken[Tiab] OR chickens[Tiab] OR gallus[Tiab] OR quail[Tiab] OR bird[Tiab] OR birds[Tiab] OR quails[Tiab] OR poultry[Tiab] OR poultries[Tiab] OR fowl[Tiab] OR fowls[Tiab] OR reptile[Tiab] OR reptilia[Tiab] OR reptiles[Tiab] OR snakes[Tiab] OR snake[Tiab] OR lizard[Tiab] OR lizards[Tiab] OR alligator[Tiab] OR alligators[Tiab] OR crocodile[Tiab] OR crocodiles[Tiab] OR turtle[Tiab] OR turtles[Tiab] OR amphibian[Tiab] OR amphibians[Tiab] OR amphibia[Tiab] OR frog[Tiab] OR frogs[Tiab] OR bombina[Tiab] OR salientia[Tiab] OR toad[Tiab] OR toads[Tiab] OR "epidalea calamita"[Tiab] OR salamander[Tiab] OR salamanders[Tiab] OR eel[Tiab] OR eels[Tiab] OR fish[Tiab] OR fishes[Tiab] OR pisces[Tiab] OR catfish[Tiab] OR catfishes[Tiab] OR siluriformes[Tiab] OR arius[Tiab] OR heteropneustes[Tiab] OR sheatfish[Tiab] OR perch[Tiab] OR perches[Tiab] OR percidae[Tiab] OR perca[Tiab] OR trout[Tiab] OR trouts[Tiab] OR char[Tiab] OR chars[Tiab] OR salvelinus[Tiab] OR "fathead minnow"[Tiab] OR minnow[Tiab] OR cyprinidae[Tiab] OR carps[Tiab] OR carp[Tiab] OR zebrafish[Tiab] OR zebrafishes[Tiab] OR goldfish[Tiab] OR goldfishes[Tiab] OR guppy[Tiab] OR guppies[Tiab] OR chub[Tiab] OR chubs[Tiab] OR tinca[Tiab] OR barbels[Tiab] OR barbus[Tiab] OR pimephales[Tiab] OR promelas[Tiab] OR "poecilia reticulata"[Tiab] OR mullet[Tiab] OR mullets[Tiab] OR seahorse[Tiab] OR seahorses[Tiab] OR mugil curema[Tiab] OR atlantic cod[Tiab] OR shark[Tiab] OR sharks[Tiab] OR catshark[Tiab] OR anguilla[Tiab] OR salmonid[Tiab] OR salmonids[Tiab] OR whitefish[Tiab] OR whitefishes[Tiab] OR salmon[Tiab] OR salmons[Tiab] OR sole[Tiab] OR solea[Tiab] OR "sea lamprey"[Tiab] OR lamprey[Tiab] OR lampreys[Tiab] OR pumpkinseed[Tiab] OR sunfish[Tiab] OR sunfishes[Tiab] OR tilapia[Tiab] OR tilapias[Tiab] OR turbot[Tiab] OR turbots[Tiab] OR flatfish[Tiab] OR flatfishes[Tiab] OR sciuridae[Tiab] OR squirrel[Tiab] OR squirrels[Tiab] OR chipmunk[Tiab] OR chipmunks[Tiab] OR suslik[Tiab] OR susliks[Tiab] OR vole[Tiab] OR voles[Tiab] OR lemming[Tiab] OR lemmings[Tiab] OR muskrat[Tiab] OR muskrats[Tiab] OR lemmus[Tiab] OR otter[Tiab] OR otters[Tiab] OR marten[Tiab] OR martens[Tiab] OR martes[Tiab] OR weasel[Tiab] OR badger[Tiab] OR badgers[Tiab] OR ermine[Tiab] OR mink[Tiab] OR minks[Tiab] OR sable[Tiab] OR sables[Tiab] OR gulo[Tiab] OR gulos[Tiab] OR wolverine[Tiab] OR wolverines[Tiab] OR minks[Tiab] OR mustela[Tiab] OR llama[Tiab] OR llamas[Tiab] OR alpaca[Tiab] OR alpacas[Tiab] OR camelid[Tiab] OR camelids[Tiab] OR guanaco[Tiab] OR guanacos[Tiab] OR chiroptera[Tiab] OR chiropteras[Tiab] OR bat[Tiab] OR bats[Tiab] OR fox[Tiab] OR foxes[Tiab] OR iguana[Tiab] OR iguanas[Tiab] OR xenopus laevis[Tiab] OR parakeet[Tiab] OR parakeets[Tiab] OR parrot[Tiab] OR parrots[Tiab] OR donkey[Tiab] OR donkeys[Tiab] OR mule[Tiab] OR mules[Tiab] OR zebra[Tiab] OR zebras[Tiab] OR shrew[Tiab] OR shrews[Tiab] OR bison[Tiab] OR bisons[Tiab] OR buffalo[Tiab] OR buffaloes[Tiab] OR deer[Tiab] OR deers[Tiab] OR bear[Tiab] OR bears[Tiab] OR panda[Tiab] OR pandas[Tiab] OR "wild hog"[Tiab] OR "wild boar"[Tiab] OR fitchew[Tiab] OR fitch[Tiab] OR beaver[Tiab] OR beavers[Tiab] OR jerboa[Tiab] OR jerboas[Tiab] OR capybara[Tiab] OR capybaras[Tiab]) NOT medline[subset]) NOT ("review"[Publication Type] OR "hascommenton"[All Fields] OR "editorial" [Publication Type] OR "letter" [Publication Type]) AND (fft[Filter]) AND (2010:2024[pdat]) AND ("English"[Language]))
PD and CR human studies (excluding clinical trials, study protocols and any animal models)	(("Parkinson disease"[Mesh] OR "Idiopathic Parkinson's Disease"[tiab] OR "Parkinson's Disease, Lewy Body"[tiab] OR "Primary Parkinsonism"[tiab] OR "Paralysis Agitans"[tiab]) AND ("Circadian rhythm"[Mesh] OR "circadian rhythm"[tiab] OR "Circadian Clock"[tiab] OR "Clock, Circadian"[tiab] OR "Clocks, Circadian"[tiab] OR "Clocks, Circadian"[tiab] OR "Circadian Clock System*"[tiab] OR "Circadian System"[tiab] OR "Circadian Timing System*"[tiab] OR "Circadian Rhythm*"[tiab] OR "Twenty-Four Hour Rhythm"[tiab] OR "Twenty Four Hour Rhythm*"[tiab] OR "Nyctohemeral Rhythm*"[tiab] OR "Nycthemeral Rhythm*"[tiab] OR "Diurnal Rhythm"[tiab] OR "Rhythms, Diurnal"[tiab]) NOT ("animals" [Mesh] NOT "humans"[Mesh]) NOT ("animal experimentation"[MeSH Terms] OR "models, animal"[MeSH Terms] OR "invertebrates"[MeSH Terms] OR "Animals"[Mesh:noexp] OR "animal population groups"[MeSH Terms] OR "chordata"[MeSH Terms:noexp] OR "chordata, nonvertebrate"[MeSH Terms] OR "vertebrates"[MeSH Terms:noexp] OR "amphibians"[MeSH Terms] OR "birds"[MeSH Terms] OR "fishes"[MeSH Terms] OR "reptiles"[MeSH Terms] OR "mammals"[MeSH Terms:noexp] OR "primates"[MeSH Terms:noexp] OR "artiodactyla"[MeSH Terms] OR "carnivora"[MeSH Terms] OR "cetacea"[MeSH Terms] OR "chiroptera"[MeSH Terms] OR "elephants"[MeSH Terms] OR "hyraxes"[MeSH Terms] OR “insectivora”[MeSH Terms] OR "lagomorpha"[MeSH Terms] OR "marsupialia"[MeSH Terms] OR "monotremata"[MeSH Terms] OR "perissodactyla"[MeSH Terms] OR "rodentia"[MeSH Terms] OR "scandentia"[MeSH Terms] OR "sirenia"[MeSH Terms] OR "xenarthra"[MeSH Terms] OR "haplorhini"[MeSH Terms:noexp] OR "strepsirhini"[MeSH Terms] OR "platyrrhini"[MeSH Terms] OR "tarsii"[MeSH Terms] OR "catarrhini"[MeSH Terms:noexp] OR "cercopithecidae"[MeSH Terms] OR "hylobatidae"[MeSH Terms] OR "hominidae"[MeSH Terms:noexp] OR "gorilla gorilla"[MeSH Terms] OR "pan paniscus"[MeSH Terms] OR "pan troglodytes"[MeSH Terms] OR "pongo pygmaeus"[MeSH Terms]) NOT ((animals[tiab] OR animal[tiab] OR mice[Tiab] OR mus[Tiab] OR mouse[Tiab] OR murine[Tiab] OR woodmouse[tiab] OR rats[Tiab] OR rat[Tiab] OR murinae[Tiab] OR muridae[Tiab] OR cottonrat[tiab] OR cottonrats[tiab] OR hamster[tiab] OR hamsters[tiab] OR cricetinae[tiab] OR rodentia[Tiab] OR rodent[Tiab] OR rodents[Tiab] OR pigs[Tiab] OR pig[Tiab] OR swine[tiab] OR swines[tiab] OR piglets[tiab] OR piglet[tiab] OR boar[tiab] OR boars[tiab] OR "sus scrofa"[tiab] OR ferrets[tiab] OR ferret[tiab] OR polecat[tiab] OR polecats[tiab] OR "mustela putorius"[tiab] OR "guinea pigs"[Tiab] OR "guinea pig"[Tiab] OR cavia[Tiab] OR callithrix[Tiab] OR marmoset[Tiab] OR marmosets[Tiab] OR cebuella[Tiab] OR hapale[Tiab] OR octodon[Tiab] OR chinchilla[Tiab] OR chinchillas[Tiab] OR gerbillinae[Tiab] OR gerbil[Tiab] OR gerbils[Tiab] OR jird[Tiab] OR jirds[Tiab] OR merione[Tiab] OR meriones[Tiab] OR rabbits[Tiab] OR rabbit[Tiab] OR hares[Tiab] OR hare[Tiab] OR diptera[Tiab] OR flies[Tiab] OR fly[Tiab] OR dipteral[Tiab] OR drosphila[Tiab] OR drosophilidae[Tiab] OR cats[Tiab] OR cat[Tiab] OR carus[Tiab] OR felis[Tiab] OR nematoda[Tiab] OR nematode[Tiab] OR nematoda[Tiab] OR nematode[Tiab] OR nematodes[Tiab] OR sipunculida[Tiab] OR dogs[Tiab] OR dog[Tiab] OR canine[Tiab] OR canines[Tiab] OR canis[Tiab] OR sheep[Tiab] OR sheeps[Tiab] OR mouflon[Tiab] OR mouflons[Tiab] OR ovis[Tiab] OR goats[Tiab] OR goat[Tiab] OR capra[Tiab] OR capras[Tiab] OR rupicapra[Tiab] OR chamois[Tiab] OR haplorhini[Tiab] OR monkey[Tiab] OR monkeys[Tiab] OR anthropoidea[Tiab] OR anthropoids[Tiab] OR saguinus[Tiab] OR tamarin[Tiab] OR tamarins[Tiab] OR leontopithecus[Tiab] OR hominidae[Tiab] OR ape[Tiab] OR apes[Tiab] OR pan[Tiab] OR paniscus[Tiab] OR "pan paniscus"[Tiab] OR bonobo[Tiab] OR bonobos[Tiab] OR troglodytes[Tiab] OR "pan troglodytes"[Tiab] OR gibbon[Tiab] OR gibbons[Tiab] OR siamang[Tiab] OR siamangs[Tiab] OR nomascus[Tiab] OR symphalangus[Tiab] OR chimpanzee[Tiab] OR chimpanzees[Tiab] OR prosimians[Tiab] OR "bush baby"[Tiab] OR prosimian[Tiab] OR bush babies[Tiab] OR galagos[Tiab] OR galago[Tiab] OR pongidae[Tiab] OR gorilla[Tiab] OR gorillas[Tiab] OR pongo[Tiab] OR pygmaeus[Tiab] OR "pongo pygmaeus"[Tiab] OR orangutans[Tiab] OR pygmaeus[Tiab] OR lemur[Tiab] OR lemurs[Tiab] OR lemuridae[Tiab] OR horse[Tiab] OR horses[Tiab] OR pongo[Tiab] OR equus[Tiab] OR cow[Tiab] OR calf[Tiab] OR bull[Tiab] OR chicken[Tiab] OR chickens[Tiab] OR gallus[Tiab] OR quail[Tiab] OR bird[Tiab] OR birds[Tiab] OR quails[Tiab] OR poultry[Tiab] OR poultries[Tiab] OR fowl[Tiab] OR fowls[Tiab] OR reptile[Tiab] OR reptilia[Tiab] OR reptiles[Tiab] OR snakes[Tiab] OR snake[Tiab] OR lizard[Tiab] OR lizards[Tiab] OR alligator[Tiab] OR alligators[Tiab] OR crocodile[Tiab] OR crocodiles[Tiab] OR turtle[Tiab] OR turtles[Tiab] OR amphibian[Tiab] OR amphibians[Tiab] OR amphibia[Tiab] OR frog[Tiab] OR frogs[Tiab] OR bombina[Tiab] OR salientia[Tiab] OR toad[Tiab] OR toads[Tiab] OR "epidalea calamita"[Tiab] OR salamander[Tiab] OR salamanders[Tiab] OR eel[Tiab] OR eels[Tiab] OR fish[Tiab] OR fishes[Tiab] OR pisces[Tiab] OR catfish[Tiab] OR catfishes[Tiab] OR siluriformes[Tiab] OR arius[Tiab] OR heteropneustes[Tiab] OR sheatfish[Tiab] OR perch[Tiab] OR perches[Tiab] OR percidae[Tiab] OR perca[Tiab] OR trout[Tiab] OR trouts[Tiab] OR char[Tiab] OR chars[Tiab] OR salvelinus[Tiab] OR "fathead minnow"[Tiab] OR minnow[Tiab] OR cyprinidae[Tiab] OR carps[Tiab] OR carp[Tiab] OR zebrafish[Tiab] OR zebrafishes[Tiab] OR goldfish[Tiab] OR goldfishes[Tiab] OR guppy[Tiab] OR guppies[Tiab] OR chub[Tiab] OR chubs[Tiab] OR tinca[Tiab] OR barbels[Tiab] OR barbus[Tiab] OR pimephales[Tiab] OR promelas[Tiab] OR "poecilia reticulata"[Tiab] OR mullet[Tiab] OR mullets[Tiab] OR seahorse[Tiab] OR seahorses[Tiab] OR mugil curema[Tiab] OR atlantic cod[Tiab] OR shark[Tiab] OR sharks[Tiab] OR catshark[Tiab] OR anguilla[Tiab] OR salmonid[Tiab] OR salmonids[Tiab] OR whitefish[Tiab] OR whitefishes[Tiab] OR salmon[Tiab] OR salmons[Tiab] OR sole[Tiab] OR solea[Tiab] OR "sea lamprey"[Tiab] OR lamprey[Tiab] OR lampreys[Tiab] OR pumpkinseed[Tiab] OR sunfish[Tiab] OR sunfishes[Tiab] OR tilapia[Tiab] OR tilapias[Tiab] OR turbot[Tiab] OR turbots[Tiab] OR flatfish[Tiab] OR flatfishes[Tiab] OR sciuridae[Tiab] OR squirrel[Tiab] OR squirrels[Tiab] OR chipmunk[Tiab] OR chipmunks[Tiab] OR suslik[Tiab] OR susliks[Tiab] OR vole[Tiab] OR voles[Tiab] OR lemming[Tiab] OR lemmings[Tiab] OR muskrat[Tiab] OR muskrats[Tiab] OR lemmus[Tiab] OR otter[Tiab] OR otters[Tiab] OR marten[Tiab] OR martens[Tiab] OR martes[Tiab] OR weasel[Tiab] OR badger[Tiab] OR badgers[Tiab] OR ermine[Tiab] OR mink[Tiab] OR minks[Tiab] OR sable[Tiab] OR sables[Tiab] OR gulo[Tiab] OR gulos[Tiab] OR wolverine[Tiab] OR wolverines[Tiab] OR minks[Tiab] OR mustela[Tiab] OR llama[Tiab] OR llamas[Tiab] OR alpaca[Tiab] OR alpacas[Tiab] OR camelid[Tiab] OR camelids[Tiab] OR guanaco[Tiab] OR guanacos[Tiab] OR chiroptera[Tiab] OR chiropteras[Tiab] OR bat[Tiab] OR bats[Tiab] OR fox[Tiab] OR foxes[Tiab] OR iguana[Tiab] OR iguanas[Tiab] OR xenopus laevis[Tiab] OR parakeet[Tiab] OR parakeets[Tiab] OR parrot[Tiab] OR parrots[Tiab] OR donkey[Tiab] OR donkeys[Tiab] OR mule[Tiab] OR mules[Tiab] OR zebra[Tiab] OR zebras[Tiab] OR shrew[Tiab] OR shrews[Tiab] OR bison[Tiab] OR bisons[Tiab] OR buffalo[Tiab] OR buffaloes[Tiab] OR deer[Tiab] OR deers[Tiab] OR bear[Tiab] OR bears[Tiab] OR panda[Tiab] OR pandas[Tiab] OR "wild hog"[Tiab] OR "wild boar"[Tiab] OR fitchew[Tiab] OR fitch[Tiab] OR beaver[Tiab] OR beavers[Tiab] OR jerboa[Tiab] OR jerboas[Tiab] OR capybara[Tiab] OR capybaras[Tiab]) NOT medline[subset]) NOT ("review"[Publication Type] OR "hascommenton"[All Fields] OR "editorial" [Publication Type] OR "letter" [Publication Type]) NOT (("clinical"[ti] AND "trial"[ti]) OR ("study"[ti] AND "protocol"[ti]) OR "clinical trial"[Publication Type] OR "randomized controlled trial"[Publication Type]) AND (fft[Filter]) AND (2010:2024[pdat]) AND ("English"[Language]))
PD and CR (only clinical trials)	("Parkinson disease"[Mesh] OR "Idiopathic Parkinson's Disease"[tiab] OR "Parkinson's Disease, Idiopathic"[tiab] OR "Parkinson's Disease, Lewy Body"[tiab] OR "Parkinson Disease, Idiopathic"[tiab] OR "Parkinson's Disease"[tiab] OR "Idiopathic Parkinson Disease"[tiab] OR "Lewy Body Parkinson Disease"[tiab] OR "Primary Parkinsonism"[tiab] OR "Parkinsonism, Primary"[tiab] OR "Paralysis Agitans"[tiab]) AND ("Circadian rhythm"[Mesh] OR "circadian rhythm"[tiab] OR "Circadian Clock"[tiab] OR "Clock, Circadian"[tiab] OR "Clocks, Circadian"[tiab] OR "Clocks, Circadian"[tiab] OR "Circadian Clock System*"[tiab] OR "Circadian System"[tiab] OR "Circadian Timing System*"[tiab] OR "Circadian Rhythm*"[tiab] OR "Twenty-Four Hour Rhythm"[tiab] OR "Twenty Four Hour Rhythm*"[tiab] OR "Nyctohemeral Rhythm*"[tiab] OR "Nycthemeral Rhythm*"[tiab] OR "Diurnal Rhythm"[tiab] OR "Rhythms, Diurnal"[tiab]) NOT ("review"[Publication Type] OR "hascommenton"[All Fields] OR "editorial" [Publication Type] OR "letter" [Publication Type]) AND (("clinical"[ti] AND "trial"[ti]) OR "clinical trial"[Publication Type] OR "randomized controlled trial"[Publication Type]) AND (fft[Filter]) AND (2010:2024[pdat]) AND ("English"[Language])
PD clinical trials (excluding circadian and PD clinical trials)	("Parkinson disease"[Mesh] OR "Idiopathic Parkinson's Disease"[tiab] OR "Parkinson's Disease, Idiopathic"[tiab] OR "Parkinson's Disease, Lewy Body"[tiab] OR "Parkinson Disease, Idiopathic"[tiab] OR "Parkinson's Disease"[tiab] OR "Idiopathic Parkinson Disease"[tiab] OR "Lewy Body Parkinson Disease"[tiab] OR "Primary Parkinsonism"[tiab] OR "Parkinsonism, Primary"[tiab] OR "Paralysis Agitans"[tiab]) NOT (("Parkinson disease"[Mesh] OR "Idiopathic Parkinson's Disease"[tiab] OR "Parkinson's Disease, Idiopathic"[tiab] OR "Parkinson's Disease, Lewy Body"[tiab] OR "Parkinson Disease, Idiopathic"[tiab] OR "Parkinson's Disease"[tiab] OR "Idiopathic Parkinson Disease"[tiab] OR "Lewy Body Parkinson Disease"[tiab] OR "Primary Parkinsonism"[tiab] OR "Parkinsonism, Primary"[tiab] OR "Paralysis Agitans"[tiab]) AND ("Circadian rhythm"[Mesh] OR "circadian rhythm"[tiab] OR "Circadian Clock"[tiab] OR "Clock, Circadian"[tiab] OR "Clocks, Circadian"[tiab] OR "Clocks, Circadian"[tiab] OR "Circadian Clock System*"[tiab] OR "Circadian System"[tiab] OR "Circadian Timing System*"[tiab] OR "Circadian Rhythm*"[tiab] OR "Twenty-Four Hour Rhythm"[tiab] OR "Twenty Four Hour Rhythm*"[tiab] OR "Nyctohemeral Rhythm*"[tiab] OR "Nycthemeral Rhythm*"[tiab] OR "Diurnal Rhythm"[tiab] OR "Rhythms, Diurnal"[tiab])) NOT (Review[Publication Type]) NOT ("review"[Publication Type] OR "hascommenton"[All Fields] OR "editorial" [Publication Type] OR "letter" [Publication Type]) AND (("clinical"[ti] AND "trial"[ti]) OR "clinical trial"[Publication Type] OR "randomized controlled trial"[Publication Type]) AND (fft[Filter]) AND (2010:2024[pdat]) AND ("English"[Language])

## Perspectives and conclusions

Neurodegenerative disorders, including PD, constitute a significant and growing challenge in our ageing global population. As we deepen our understanding of the molecular mechanisms underlying PD, it becomes increasingly clear that the circadian clock plays a pivotal role in the disease’s pathophysiology. Circadian disruptions not only exacerbate motor and nonmotor symptoms but also potentially accelerate disease progression by influencing key cellular processes such as mitochondrial function, oxidative stress, and neuroinflammation. Sleep problems (e.g., REM sleep behavior disorder), altered hormonal secretion, and disrupted core body temperature rhythms, emerge years before motor symptoms, as we also discussed in this review and could be used for the development of markers for this prodromal stage. Molecular evidence also strongly links circadian clock dysfunction to neurodegeneration, particularly through disruptions in core-clock genes (e.g., *BMAL1* and *PER2*), and clock-controlled genes, which play critical roles in cellular homeostasis, mitochondrial function, and neuroinflammation. Furthermore, interventions to revert circadian changes, including BLT or melatonin supplements, have shown promising benefits in improving both motor and nonmotor symptoms. Thus, if circadian disruption were purely a consequence of PD, the observed benefits of circadian-based interventions would be less likely, suggesting a bidirectional relationship where circadian dysfunction may, in addition, accelerate disease onset and or progression, as well as symptoms. This review has highlighted the relevance of CR in PD, suggesting that time-of-day factors should be considered in both the research and clinical management of this condition.

Current PD management strategies primarily focus on symptomatic relief through pharmacological and surgical interventions. However, these approaches often overlook the importance of circadian alignment, which could offer a complementary avenue for improving treatment outcomes. Incorporating circadian-based therapies, such as timed light exposure, exercise, and meal timing, into treatment plans may enhance the efficacy of existing interventions, reduce side effects, and improve the overall quality of life of PD patients.

Looking forward, further research is needed to elucidate the exact mechanisms by which circadian disruptions contribute to PD and to develop personalized chronotherapeutic strategies. Future large-scale clinical trials should investigate the timing of drug administration and other pharmacological or non-pharmacological interventions to align with the individual circadian profiles of PD patients. Additionally, the exploration of biomarkers related to circadian rhythms could lead to earlier diagnosis and more precise monitoring of disease progression.

Hence, recognizing and integrating the circadian dimension in PD research and management holds great promise for advancing our understanding of the disease and enhancing the well-being of those affected. As we continue to explore this frontier, it is crucial to foster interdisciplinary collaborations that bridge the gap between chronobiology and neurology, ultimately paving the way for innovative treatments and improved patient care.

## Data Availability

No datasets were generated or analysed during the current study.
